# Di­ammonium potassium citrate, (NH_4_)_2_KC_6_H_5_O_7_

**DOI:** 10.1107/S2414314620006124

**Published:** 2020-05-12

**Authors:** Nilan V. Patel, Joseph T. Golab, James A. Kaduk

**Affiliations:** a Illinois Mathematics and Science Academy, 1500 Sullivan Road, Aurora IL 60506 , USA; b Department of Chemistry, North Central College, 131 S. Loomis, St., Naperville IL, 60540 , USA; University of Aberdeen, Scotland

**Keywords:** powder diffraction, density functional theory, citrate anion, ammonium, potasssium

## Abstract

The crystal structure of di­ammonium potassium citrate, 2NH_4_^+^·K^+^·C_6_H_5_O_7_^3−^, has been solved and refined using laboratory X-ray powder diffraction data and optimized using density functional theory. The KO_7_ coordination polyhedra are isolated. The ammonium cations and the hydro­phobic methyl­ene sides of the citrate anions occupy the spaces between the coordination polyhedra. Each hydrogen atom of the ammonium ions acts as a donor in a charge-assisted N—H⋯O, N—H⋯(O,O) or N—H⋯(O,O,O) hydrogen bond. There is an intra­molecular O—H⋯O hydrogen bond in the citrate anion between the hydroxide group and one of the terminal carboxyl­ate groups.

## Abstract 2

The crystal structure of di­ammonium potassium citrate has been solved and refined using laboratory X-ray powder diffraction data and optimized using density functional theory techniques.

## Graphical abstract

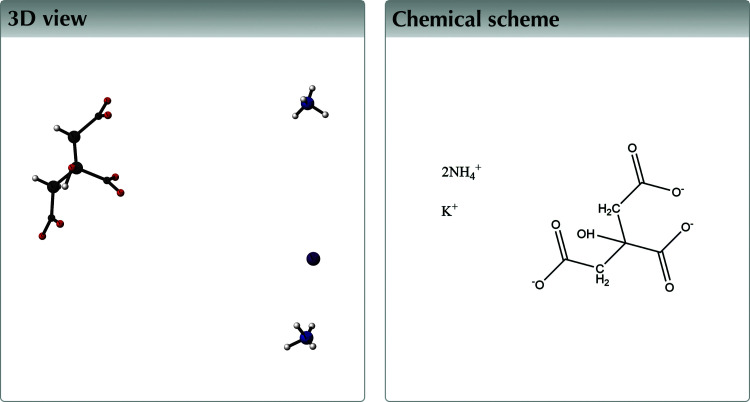

## Structure description

A systematic study of the crystal structures of Group 1 (alkali metal) citrate salts has been reported by Rammohan & Kaduk (2018 ▸). The study was extended to ammonium citrates by Wheatley & Kaduk (2019 ▸). The title compound represents a further extension to mixed ammonium Group 1 citrates, specifically di­ammonium potassium citrate, (NH_4_)_2_KC_6_H_5_O_7_.

**Figure 1:**
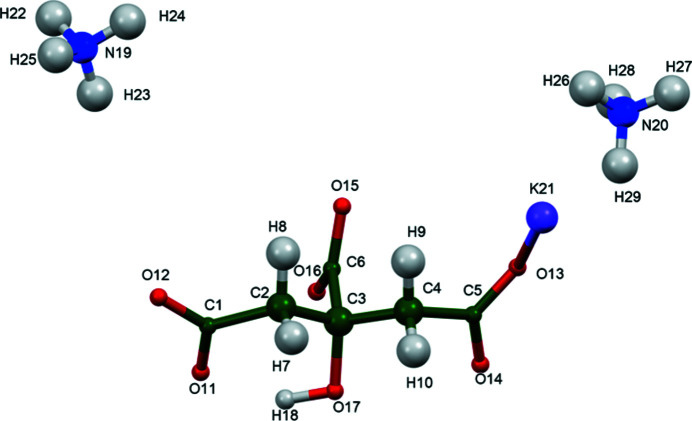
The asymmetric unit of (NH_4_)_2_KC_6_H_5_O_7_ with the atom numbering and 50% probability spheroids.

**Figure 2:**
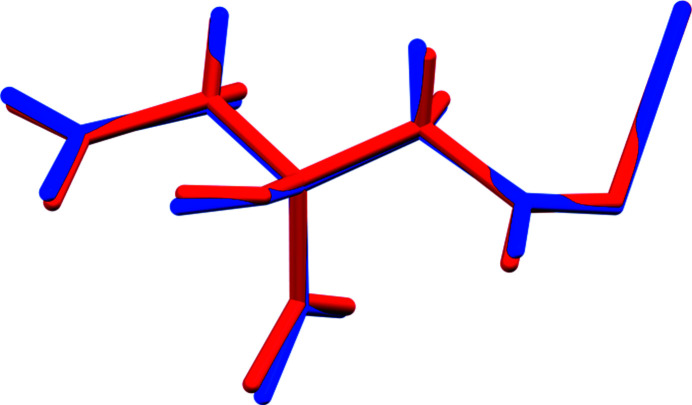
Comparison of the refined and optimized structures of (NH_4_)_2_KC_6_H_5_O_7_. The refined structure is in red, and the DFT-optimized structure is in blue.

The structure of (NH_4_)_2_KC_6_H_5_O_7_ was solved and refined from powder X-ray data and optimized by density functional theory (DFT) calculations (see *Experimental* section) and is illustrated in Fig. 1 ▸. The root-mean-square Cartesian displacement of the non-hydrogen citrate atoms in the Rietveld-refined and DFT-optimized structures is 0.108 Å (Fig. 2 ▸). The maximum deviation is 0.211 Å, at O14. The r.m.s. displacement of the potassium ions is 0.054 Å. The r.m.s. displacements of the ammonium ions N19 and N20 are 0.111 and 0.151 Å respectively. The good agreement between the two structures is strong evidence that the experimental structure is correct (van de Streek & Neumann, 2014 ▸). All of the citrate bond distances, bond angles, and torsion angles fall within the normal ranges indicated by a *Mercury Mogul* Geometry Check (Macrae *et al.*, 2020 ▸). The citrate anion occurs in the *trans,trans*-conformation (about C2—C3 and C3—C4), which is one of the two low-energy conformations of an isolated citrate anion (Rammohan & Kaduk, 2018 ▸). The central carboxyl­ate group and the hydroxyl group exhibit a small twist [O16—C6—C3—O17 torsion angle = 7.0°] from the normal planar arrangement. The Mulliken overlap populations indicate that the K—O bonds are ionic.

The citrate anion doubly chelates to K21 through the hydroxyl group O17 and the terminal carboxyl­ate group (atom O11). The anion doubly chelates to another potassium cation through the hydroxyl group and the other terminal carboxyl­ate group (atom O14). Each oxygen atom bonds to a single potassium cation. As a result, K21 is seven-coordinate (capped trigonal prismatic), with a bond-valence sum of 0.98.

The Bravais–Friedel–Donnay–Harker (Bravais, 1866 ▸; Friedel, 1907 ▸; Donnay & Harker, 1937 ▸) method suggests that we might expect block morphology for di­ammonium potassium citrate. A 2nd order spherical harmonic preferred orientation model was included in the Rietveld refinement; the texture index was 1.179, indicating that preferred orientation was significant for this rotated flat sheet specimen.

**Figure 3:**
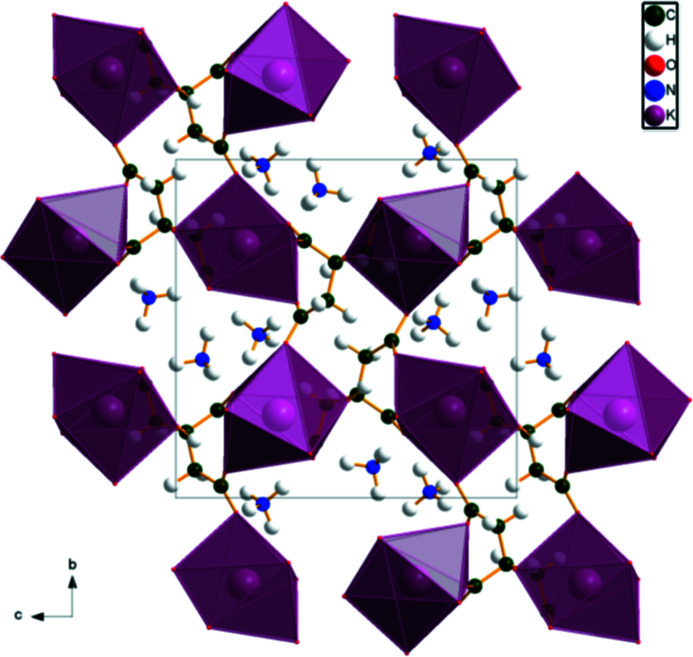
The crystal structure of (NH_4_)_2_KC_6_H_5_O_7_, viewed along the *a* axis.

The KO_7_ coordination polyhedra are isolated (Fig. 3 ▸). The ammonium cations and the hydro­phobic methyl­ene sides of the citrate anions occupy the spaces between the coordination polyhedra. Each hydrogen atom of the ammonium ions acts as a donor in a charge-assisted N—H⋯O hydrogen bond; there is one bifurcated *M*—H⋯(O,O) bond and one trifurcated N—H⋯(O,O,O) bond (Table 1 ▸). There is an intra­molecular hydrogen bond between the hydroxide group and one of the terminal carboxyl­ate groups. The N—H⋯O hydrogen-bond energies were calculated by the correlation of Wheatley & Kaduk (2019 ▸), and the O—H⋯O hydrogen bond energy was calculated by the correlation of Rammohan & Kaduk (2018 ▸).

**Figure 4:**
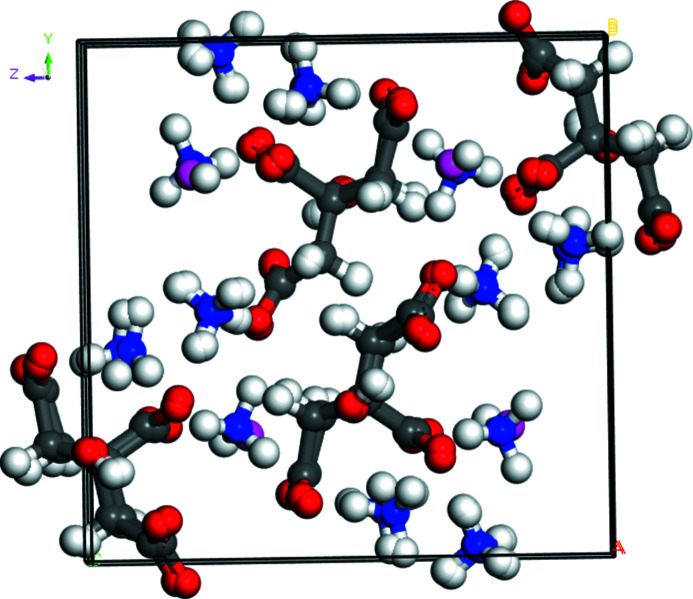
Overlay of the crystal structures of di­ammonium potassium citrate and tri­ammonium citrate, showing that they are isostructural.

**Figure 5:**
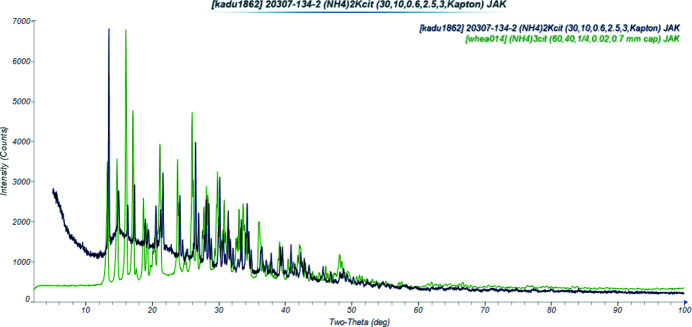
Comparison of the X-ray powder diffraction patterns of di­ammonium potassium citrate (black) and tri­ammonium citrate (green).

Di­ammonium potassium citrate is isostructural to trimmonium citrate (Wheatley & Kaduk, 2019 ▸; Fig. 4 ▸). Comparison of the powder patterns (Fig. 5 ▸) confirms the similarity.

Details of the comprehensive literature search for citrate structures are presented in Rammohan & Kaduk (2018 ▸). The powder pattern of (NH_4_)_2_KC_6_H_5_O_7_ was indexed using *N-TREOR* (Altomare *et al.*, 2013 ▸). A reduced-cell search of the cell of di­ammonium potassium citrate in the Cambridge Structural Database (Groom *et al.*, 2016 ▸) resulted in no hits.

## Synthesis and crystallization

Di­ammonium potassium citrate was synthesized by dissolving 1.1217 g di­ammonium hydrogen citrate (Fisher Lot #995047) and 0.3279 g potassium carbonate (Sigma–Aldrich Lot #098 K0064) in ∼5 ml of deionized water. The clear solution was dried at 363 K for two days to yield a white solid.

## Refinement

**Figure 6:**
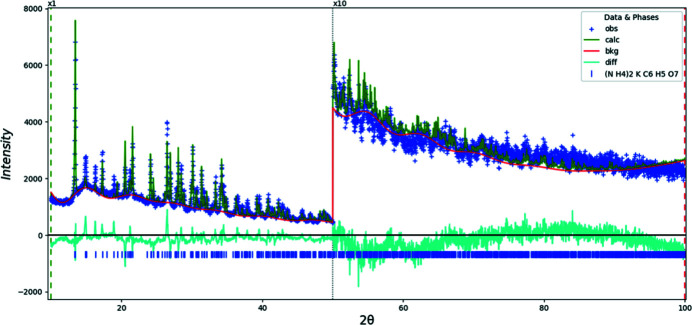
Rietveld plot for (NH_4_)_2_KC_6_H_5_O_7_. The blue crosses represent the observed data points, and the green line is the calculated pattern. The cyan curve is the normalized error plot. The vertical scale has been multiplied by a factor of 10× for 2θ > 50.0°. The row of blue tick marks indicates the calculated reflection positions. The red line is the background curve.

Crystal data, data collection and structure refinement details are summarized in Table 2 ▸. A Rietveld plot is presented in Fig. 6 ▸.

The structure was solved using Monte Carlo simulated annealing techniques with *FOX* (Favre-Nicolin & Černý 2002 ▸) using a citrate anion, one K^+^ cation and two ammonium cations as fragments. The structure was refined by the Rietveld method using *GSAS-II* (Toby & Von Dreele, 2013 ▸). The hydrogen atoms were included in fixed positions, which were recalculated during the course of the refinement using *Materials Studio* (Dassault Systems, 2019 ▸). All C—C and C—O bond distances and all bond angles were restrained based on a *Mercury*/*Mogul* Geometry Check (Sykes *et al.*, 2011 ▸; Bruno *et al.*, 2004 ▸) of the mol­ecule. The *U*_iso_ values of the atoms in the central and outer portions of the citrate were constrained to be equal, and the *U*_iso_ values of the hydrogen atoms were constrained to be 1.3× those of the atoms to which they are attached. A Chebyschev background function with three coefficients was used to model the background. A ten-term diffuse scattering function was used to describe the scattering from the capillary and any amorphous component. A density functional geometry optimization was carried out using *CRYSTAL14* (Dovesi *et al.*, 2014 ▸). The basis sets for the H, C, N, and O atoms were those of Gatti *et al.* (1994 ▸), and the basis set for K was that of Peintinger *et al.* (2013 ▸). The calculation was run on eight 2.1 GHz Xeon cores (each with 6 Gb RAM) of a 304-core Dell Linux cluster at IIT, using 8 *k*-points and the B3LYP functional, and took ∼5 days.

## Supplementary Material

Crystal structure: contains datablock(s) global, I, I_DFT. DOI: 10.1107/S2414314620006124/hb4336sup1.cif

Supporting information file. DOI: 10.1107/S2414314620006124/hb4336Isup2.cml

CCDC references: 2001358, 2001359

Additional supporting information:  crystallographic information; 3D view; checkCIF report

## Figures and Tables

**Table 1:** Hydrogen-bond geometry (Å, °)

*D*—H⋯*A*	*D*—H	H⋯*A*	*D*⋯*A*	*D*—H⋯*A*
O17—H18⋯O11	0.90	1.79	2.613 (19)	150
N19—H22⋯O14^i^	0.77	2.18	2.88 (3)	150
N19—H22⋯O16^i^	0.77	2.46	2.80 (3)	109
N19—H22⋯O17^i^	0.77	2.33	2.74 (2)	114
N19—H23⋯O16^i^	0.97	2.13	2.80 (3)	125
N19—H24⋯O15^ii^	0.85	2.13	2.90 (3)	151
N19—H25⋯O14^iii^	1.07	1.77	2.80 (2)	162
N20—H26⋯O15^iv^	0.94	2.58	3.26 (3)	130
N20—H26⋯O16^iv^	0.94	1.91	2.84 (3)	173
N20—H27⋯O11^v^	0.92	1.78	2.69 (3)	177
N20—H28⋯O13^vi^	0.98	1.99	2.89 (3)	154
N20—H29⋯O12^vii^	0.86	1.92	2.77 (3)	170

Symmetry codes: (i) 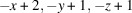 ; (ii) 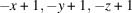 ; (iii) 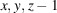 ; (iv) 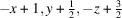 ; (v) 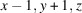 ; (vi) 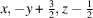 ; (vii) 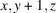 .

**Table 2:** Experimental details

Crystal data	
Chemical formula	2NH_4_^+^·K^+^·C_6_H_5_O_7_^3−^
*M*_r_	264.27
Crystal system, space group	Monoclinic, *P*2_1_/*c*
Temperature (K)	300
*a*, *b*, *c* (Å)	6.0238 (5), 13.2925 (6), 13.4155 (8)
β (°)	93.131 (4)
*V* (Å^3^)	1072.60 (12)
*Z*	4
Radiation type	*K*α_1,2_, λ = 1.54059, 1.54445 Å
Specimen shape, size (mm)	Flat sheet, 25 × 25

Data collection	
Diffractometer	Bruker D2 Phaser
Specimen mounting	Standard sample holder with Kapton window
Data collection mode	Reflection
Scan method	Step
2θ values (°)	2θ_min_ = 5.051, 2θ_max_ = 100.038, 2θ_step_ = 0.020

Refinement	
*R* factors and goodness of fit	*R*_p_ = 0.056, *R*_wp_ = 0.072, *R*_exp_ = 0.038, *R*(*F* ^2^) = 0.16190, χ^2^ = 3.656
No. of parameters	78
H-atom treatment	Only H-atom displacement parameters refined

The same symmetry and lattice parameters were used for the DFT calculations as for the powder diffraction study. Computer programs: *Data Collector* (Bruker, 2015 ▸), *FOX* (Favre-Nicolin & Černý, 2002 ▸), *GSAS-II* (Toby & Von Dreele, 2013 ▸), *DIAMOND* (Crystal Impact, 2015 ▸) and *Mercury* (Macrae *et al.*, 2020 ▸).

## full crystallographic data

### Diammonium potassium citrate (I) Crystal data

**Table:**

2NH_4_^+^·K^+^·C_6_H_5_O_7_^3^^−^	*Z* = 4
*M**_r_* = 264.27	*D*_x_ = 1.637 Mg m^−^^3^
Monoclinic, *P*2_1_/*c*	*K*α_1,2_ radiation, λ = 1.54059, 1.54445 Å
Hall symbol: -P 2ybc	*T* = 300 K
*a* = 6.0238 (5) Å	Particle morphology: powder
*b* = 13.2925 (6) Å	white
*c* = 13.4155 (8) Å	flat_sheet, 25 × 25 mm
β = 93.131 (4)°	Specimen preparation: Prepared at 363 K and 101 kPa
*V* = 1072.60 (12) Å^3^	

### Diammonium potassium citrate (I) Data collection

**Table:**

Bruker D2 Phaser diffractometer	Data collection mode: reflection
Radiation source: sealed X-ray tube	Scan method: step
Ni filter monochromator	2θ_min_ = 5.051°, 2θ_max_ = 100.038°, 2θ_step_ = 0.020°
Specimen mounting: standard sample holder with Kapton window	

### Diammonium potassium citrate (I) Refinement

**Table:**

Least-squares matrix: full	78 parameters
*R*_p_ = 0.056	3 constraints
*R*_wp_ = 0.072	Only H-atom displacement parameters refined
*R*_exp_ = 0.038	Weighting scheme based on measured s.u.'s
*R*(*F*^2^) = 0.16190	(Δ/σ)_max_ < 0.001
4700 data points	Background function: Background function: "chebyschev" function with 3 terms: 434.7(23), -456(8), 208(14), Background Debye function parameters: A, R, U: 11.0(8), 1.430, 0.100, 30(5), 2.270, 0.100, 95(21), 2.940, 0.100, -4.4(4), 8.740, 0.100, -28(3), 1.970, 0.100, -104(22), 2.880, 0.100, -8.4(18), 3.580, 0.100, 8.2(6), 14.000, 0.100, 2.5(7), 17.810, 0.100, -0.4(8), 4.000, 0.100,
Profile function: Finger-Cox-Jephcoat function parameters U, V, W, X, Y, SH/L: peak variance(Gauss) = Utan(Th)^2^+Vtan(Th)+W: peak HW(Lorentz) = X/cos(Th)+Ytan(Th); SH/L = S/L+H/L U, V, W in (centideg)^2^, X & Y in centideg 3.537, -1.411, 1.973, 2.482, 0.000, 0.048, Crystallite size in microns with "isotropic" model: parameters: Size, G/L mix 1.000, 1.000, Microstrain, "generalized" model (10^6^ * delta Q/Q) parameters: S400, S040, S004, S220, S202, S022, S301, S103, S121, G/L mix 14695.026, 1.365, 571.072, 1337.970, 183.112, -17.422, 2458.309, -70.133, 156.113, 1.000,	Preferred orientation correction: Simple spherical harmonic correction Order = 0 Coefficients:

### Diammonium potassium citrate (I) Fractional atomic coordinates and isotropic or equivalent isotropic displacement parameters (Å^2^)

**Table:**

	*x*	*y*	*z*	*U*_iso_*/*U*_eq_	
C1	0.886 (3)	0.0374 (11)	0.8584 (14)	0.012 (2)*	
C2	0.757 (2)	0.0812 (8)	0.9422 (10)	0.036 (7)*	
C3	0.7931 (14)	0.1942 (7)	0.9608 (7)	0.036*	
C4	0.669 (2)	0.2264 (7)	1.0527 (10)	0.036*	
C5	0.656 (2)	0.3389 (7)	1.0721 (15)	0.0123*	
C6	0.7043 (15)	0.2540 (13)	0.8679 (10)	0.0123*	
H7	0.77670	0.04320	0.99960	0.046*	
H8	0.57450	0.06940	0.92620	0.046*	
H9	0.50370	0.20020	1.04630	0.046*	
H10	0.71580	0.19530	1.10950	0.046*	
O12	0.807 (2)	−0.0389 (9)	0.8144 (10)	0.0123*	
O13	0.473 (2)	0.3730 (9)	1.0971 (13)	0.0123*	
O14	0.827 (2)	0.3899 (9)	1.0640 (13)	0.0123*	
O15	0.4977 (17)	0.2596 (12)	0.8525 (10)	0.0123*	
O16	0.845 (2)	0.2922 (10)	0.8155 (9)	0.0123*	
O17	1.0258 (16)	0.2132 (9)	0.9785 (9)	0.0123*	
O11	1.061 (2)	0.0808 (9)	0.8363 (12)	0.0123*	
H18	1.08650	0.16790	0.93840	0.016*	
N19	0.825 (4)	0.6005 (14)	0.0743 (17)	0.040000*	
N20	0.369 (4)	0.9754 (16)	0.7422 (14)	0.040*	
K21	0.8415 (12)	0.7585 (6)	0.3071 (5)	0.040*	
H22	0.93790	0.61560	0.05380	0.052000*	
H23	0.90790	0.60060	0.13800	0.052000*	
H24	0.69960	0.62600	0.08460	0.052000*	
H25	0.79170	0.52180	0.07060	0.052000*	
H26	0.30840	0.91370	0.72030	0.052000*	
H27	0.26010	1.01030	0.77260	0.052000*	
H28	0.38690	1.01050	0.67920	0.052000*	
H29	0.50180	0.97670	0.76900	0.052000*	

### Diammonium potassium citrate (I) Geometric parameters (Å, º)

**Table:**

C1—C2	1.520 (3)	O15—K21^iv^	2.887 (13)
C1—O12	1.254 (5)	O16—C6	1.238 (6)
C1—O11	1.249 (4)	O16—K21^v^	2.659 (15)
C2—C1	1.520 (3)	O17—C3	1.431 (4)
C2—C3	1.536 (3)	O17—H18	0.899
C2—H7	0.923	O17—K21^iii^	3.004 (14)
C2—H8	1.119	O11—C1	1.249 (4)
C3—C2	1.536 (3)	O11—K21^v^	2.956 (15)
C3—C4	1.537 (3)	H18—O17	0.899
C3—C6	1.549 (3)	N19—H22	0.774
C3—O17	1.431 (4)	N19—H23	0.966
C4—C3	1.537 (3)	N19—H24	0.847
C4—C5	1.520 (3)	N19—H25	1.065
C4—H9	1.055	N20—H26	0.937
C4—H10	0.899	N20—H27	0.916
C5—C4	1.520 (3)	N20—H28	0.977
C5—O13	1.251 (5)	N20—H29	0.861
C5—O14	1.244 (4)	K21—O12^vi^	2.928 (14)
C6—C3	1.549 (3)	K21—O13^vii^	2.799 (16)
C6—O15	1.253 (6)	K21—O14^viii^	3.108 (16)
C6—O16	1.238 (6)	K21—O15^iv^	2.887 (13)
H7—C2	0.923	K21—O16^v^	2.659 (15)
H8—C2	1.119	K21—O17^viii^	3.004 (14)
H9—C4	1.055	K21—O11^v^	2.956 (15)
H9—H10	1.4957	H22—N19	0.774
H10—C4	0.899	H23—N19	0.966
O12—C1	1.254 (5)	H24—N19	0.847
O12—K21^i^	2.928 (14)	H25—N19	1.065
O13—C5	1.251 (5)	H26—N20	0.937
O13—K21^ii^	2.799 (16)	H27—N20	0.916
O14—C5	1.244 (4)	H28—N20	0.977
O14—K21^iii^	3.108 (16)	H29—N20	0.861
O15—C6	1.253 (6)		
			
C2—C1—O12	117.5 (5)	C4—C5—O13	117.3 (5)
C2—C1—O11	118.1 (4)	C4—C5—O14	118.0 (4)
O12—C1—O11	124.4 (4)	O13—C5—O14	124.7 (4)
C1—C2—C3	114.9 (4)	C3—C6—O15	117.4 (3)
C1—C2—H7	111.1	C3—C6—O16	116.8 (3)
C3—C2—H7	112.9	O15—C6—O16	125.9 (4)
C1—C2—H8	110.0	C6—O16—K21^v^	141.1 (13)
C3—C2—H8	107.3	C3—O17—H18	102.1
H7—C2—H8	99.4	H22—N19—H23	83.8
C2—C3—C4	109.4 (4)	H22—N19—H24	139.7
C2—C3—C6	109.4 (4)	H23—N19—H24	106.1
C4—C3—C6	109.9 (4)	H22—N19—H25	113.9
C2—C3—O17	109.2 (4)	H23—N19—H25	97.5
C4—C3—O17	109.4 (4)	H24—N19—H25	103.5
C6—C3—O17	109.6 (4)	H26—N20—H27	108.0
C3—C4—C5	116.4 (5)	H26—N20—H28	102.0
C3—C4—H9	110.0	H27—N20—H28	105.1
C5—C4—H9	106.2	H26—N20—H29	119.0
C3—C4—H10	114.1	H27—N20—H29	118.4
C5—C4—H10	108.9	H28—N20—H29	101.9
H9—C4—H10	99.6		

Symmetry codes: (i) *x*, −*y*+1/2, *z*+1/2; (ii) −*x*+1, *y*−1/2, −*z*+3/2; (iii) −*x*+2, *y*−1/2, −*z*+3/2; (iv) −*x*+1, −*y*+1, −*z*+1; (v) −*x*+2, −*y*+1, −*z*+1; (vi) *x*, −*y*+1/2, *z*−1/2; (vii) −*x*+1, *y*+1/2, −*z*+3/2; (viii) −*x*+2, *y*+1/2, −*z*+3/2.

### Diammonium potassium citrate (I) Hydrogen-bond geometry (Å, º)

**Table:**

*D*—H···*A*	*D*—H	H···*A*	*D*···*A*	*D*—H···*A*
O17—H18···O11	0.90	1.79	2.613 (19)	150
N19—H22···O14^v^	0.77	2.18	2.88 (3)	150
N19—H22···O16^v^	0.77	2.46	2.80 (3)	109
N19—H22···O17^v^	0.77	2.33	2.74 (2)	114
N19—H23···O16^v^	0.97	2.13	2.80 (3)	125
N19—H24···O15^iv^	0.85	2.13	2.90 (3)	151
N19—H25···O14^ix^	1.07	1.77	2.80 (2)	162
N20—H26···O15^vii^	0.94	2.58	3.26 (3)	130
N20—H26···O16^vii^	0.94	1.91	2.84 (3)	173
N20—H27···O11^x^	0.92	1.78	2.69 (3)	177
N20—H28···O13^xi^	0.98	1.99	2.89 (3)	154
N20—H29···O12^xii^	0.86	1.92	2.77 (3)	170

Symmetry codes: (iv) −*x*+1, −*y*+1, −*z*+1; (v) −*x*+2, −*y*+1, −*z*+1; (vii) −*x*+1, *y*+1/2, −*z*+3/2; (ix) *x*, *y*, *z*−1; (x) *x*−1, *y*+1, *z*; (xi) *x*, −*y*+3/2, *z*−1/2; (xii) *x*, *y*+1, *z*.

### (I_DFT) Crystal data

**Table:**

C6H13KN2O_7_	*c* = 13.4156 Å
*M**_r_* = 264.27	β = 93.1310°
Monoclinic, *P*2_1_/*c*	*V* = 1072.60 Å^3^
Hall symbol: -P 2ybc	*Z* = 4
*a* = 6.0238 Å	*D*_x_ = 1.637 Mg m^−^^3^
*b* = 13.2926 Å	

### (I_DFT) Data collection

**Table:**

*h* = →	*l* = →
*k* = →	

### (I_DFT) Fractional atomic coordinates and isotropic or equivalent isotropic displacement parameters (Å^2^)

**Table:**

	*x*	*y*	*z*	*U*_iso_*/*U*_eq_	
C1	0.87318	0.03565	0.86687	0.01230*	
C2	0.72599	0.08314	0.94344	0.03600*	
C3	0.76903	0.19496	0.96828	0.03600*	
C4	0.63480	0.22315	1.05833	0.03600*	
C5	0.63310	0.33416	1.08863	0.01230*	
C6	0.68765	0.25946	0.87689	0.01230*	
H7	0.75607	0.03931	1.01180	0.04600*	
H8	0.55188	0.07301	0.91973	0.04600*	
H9	0.46410	0.19855	1.04385	0.04600*	
H10	0.70341	0.18054	1.12249	0.04600*	
O12	0.80347	−0.04262	0.82266	0.01230*	
O13	0.44391	0.37143	1.10579	0.01230*	
O14	0.81343	0.38194	1.09793	0.01230*	
O15	0.48252	0.26029	0.85377	0.01230*	
O16	0.83050	0.30870	0.83032	0.01230*	
O17	0.99968	0.21047	0.99448	0.01230*	
O11	1.06265	0.07558	0.85523	0.01230*	
H18	1.07924	0.17143	0.94538	0.01600*	
N20	0.37168	0.98204	0.74458	0.04000*	
K21	0.86217	0.75540	0.30100	0.04000*	
H22	0.78800	0.60364	0.00308	0.05200*	
H23	0.95796	0.62600	0.10404	0.05200*	
N19	0.81121	0.59136	1.07827	0.04000*	
H24	0.68536	0.63002	0.11046	0.05200*	
H25	0.80797	0.51452	0.09426	0.05200*	
H26	0.30306	0.91502	0.71752	0.05200*	
H27	0.25619	1.01594	0.78942	0.05200*	
H28	0.40466	1.03125	0.68656	0.05200*	
H29	0.52233	0.96762	0.78341	0.05200*	

### (I_DFT) Bond lengths (Å)

**Table:**

C1—C2	1.530	C6—O16	1.272
C1—O12	1.258	O17—H18	0.984
C1—O11	1.276	N20—H26	1.039
C2—C3	1.542	N20—H27	1.046
C2—H7	1.093	N20—H28	1.044
C2—H8	1.088	N20—H29	1.039
C3—C4	1.536	H22—N19^i^	1.024
C3—C6	1.553	H23—N19^i^	1.039
C3—O17	1.429	N19—H23^ii^	1.039
C4—C5	1.531	N19—H22^ii^	1.024
C4—H9	1.086	N19—H24^ii^	1.030
C4—H10	1.093	N19—H25^ii^	1.044
C5—O13	1.275	H24—N19^i^	1.030
C5—O14	1.259	H25—N19^i^	1.044
C6—O15	1.257		

Symmetry codes: (i) *x*, *y*, *z*−1; (ii) *x*, *y*, *z*+1.

### (I_DFT) Hydrogen-bond geometry (Å, º)

**Table:**

*D*—H···*A*	*D*—H	H···*A*	*D*···*A*	*D*—H···*A*
N20—H29···O12	1.039	1.751	2.770	165.64
N20—H28···O13	1.044	1.712	2.746	169.62
N20—H27···O11	1.046	1.697	2.742	176.08
N20—H26···O16	1.039	1.732	2.769	175.39
N19—H25···O14	1.044	1.763	2.796	159.41
N19—H24···O15	1.030	1.853	2.833	157.81
N19—H23···O16	1.039	1.741	2.764	167.24
N19—H22···O13	1.024	1.993	2.879	143.23
O17—H18···O11	0.984	1.756	2.632	146.38

## References

[bb1] Altomare, A., Cuocci, C., Giacovazzo, C., Moliterni, A., Rizzi, R., Corriero, N. & Falcicchio, A. (2013). *J. Appl. Cryst.* **46**, 1231–1235.

[bb2] Bravais, A. (1866). *Etudes Cristallographiques*. Paris: Gauthier Villars.

[bb3] Bruker (2015). *Data Collector*. Bruker AXS Inc., Maddison, Wisconsin, USA.

[bb4] Bruno, I. J., Cole, J. C., Kessler, M., Luo, J., Motherwell, W. D. S., Purkis, L. H., Smith, B. R., Taylor, R., Cooper, R. I., Harris, S. E. & Orpen, A. G. (2004). *J. Chem. Inf. Comput. Sci.* **44**, 2133–2144. 10.1021/ci049780b 15554684

[bb5] Crystal Impact (2015). *DIAMOND*. Crystal Impact GbR, Bonn, Germany.

[bb6] Dassault Systems. (2019). *Materials Studio*, BIOVIA, San Diego, USA.

[bb7] Donnay, J. D. H. & Harker, D. (1937). *Am. Mineral.* **22**, 446–467.

[bb8] Dovesi, R., Orlando, R., Erba, A., Zicovich-Wilson, C. M., Civalleri, B., Casassa, S., Maschio, L., Ferrabone, M., De La Pierre, M., D’Arco, P., Noël, Y., Causà, M., Rérat, M. & Kirtman, B. (2014). *Int. J. Quantum Chem.* **114**, 1287–1317.

[bb9] Favre-Nicolin, V. & Černý, R. (2002). *J. Appl. Cryst.* **35**, 734–743.

[bb10] Friedel, G. (1907). *Bull. Soc. Fr. Mineral.* **30**, 326–455.

[bb11] Gatti, C., Saunders, V. R. & Roetti, C. (1994). *J. Chem. Phys.* **101**, 10686–10696.

[bb12] Groom, C. R., Bruno, I. J., Lightfoot, M. P. & Ward, S. C. (2016). *Acta Cryst.* B**72**, 171–179. 10.1107/S2052520616003954 PMC4822653 27048719

[bb13] Macrae, C. F., Sovago, I., Cottrell, S. J., Galek, P. T. A., McCabe, P., Pidcock, E., Platings, M., Shields, G. P., Stevens, J. S., Towler, M. & Wood, P. A. (2020). *J. Appl. Cryst.* **53**, 226–235. 10.1107/S1600576719014092 PMC6998782 32047413

[bb14] Peintinger, M. F., Oliveira, D. V. & Bredow, T. (2013). *J. Comput. Chem.* **34**, 451–459. 10.1002/jcc.23153 23115105

[bb15] Rammohan, A. & Kaduk, J. A. (2018). *Acta Cryst.* B**74**, 239–252. 10.1107/S2052520618002330 29616997

[bb16] Streek, J. van de & Neumann, M. A. (2014). *Acta Cryst.* B**70**, 1020–1032. 10.1107/S2052520614022902 PMC4468513 25449625

[bb17] Sykes, R. A., McCabe, P., Allen, F. H., Battle, G. M., Bruno, I. J. & Wood, P. A. (2011). *J. Appl. Cryst.* **44**, 882–886. 10.1107/S0021889811014622 PMC3246811 22477784

[bb18] Toby, B. H. & Von Dreele, R. B. (2013). *J. Appl. Cryst.* **46**, 544–549.

[bb19] Wheatley, A. M. & Kaduk, J. A. (2019). *Powder Diffr.* **34**, 35–43.

